# Markerless fliC Knockout in *Pseudomonas fluorescens* and Preliminary Evaluation on *Pinus massoniana* Callus

**DOI:** 10.3390/biology15161403

**Published:** 2026-08-16

**Authors:** Haoran Liu, Sushuang Liu, Jia Wu, Xinye Wu, Jikai Ding, Liwen Wang, Yaoyu Feng, Linyuan Zhang, Yang Li

**Affiliations:** 1 College of Life Sciences, Huzhou University, Huzhou 313000, China; lhr020620@dingtalk.com (H.L.); wxyray1108@163.com (X.W.); adjkemail@163.com (J.D.); wanglw0301@163.com (L.W.); 18437373362@163.com (L.Z.); 2 School of Life and Health Sciences, Huzhou College, Huzhou 313000, China; liu@zjhzu.edu.cn; 3 Anji County Forestry Bureau, Huzhou 313300, China; wujia123666@163.com; 4 College of Forestry and Biotechnology, Zhejiang A&F University, Hangzhou 311300, China; 18647843537@163.com

**Keywords:** *Pseudomonas fluorescens*, *fliC* gene, flagellin, pine wilt disease

## Abstract

Pine wilt disease is a devastating forest disease caused by *Bursaphelenchus xylophilus* infection. The associated bacterium *Pseudomonas fluorescens* and its flagellin protein have been implicated in this process, but the role of the flagellin-encoding gene *fliC* in pathogenesis remains unclear. Current gene knockout strategies in *P. fluorescens* are limited by low transformation efficiency and cumbersome procedures, which constrain functional studies of this gene. To address this methodological gap, we established a *fliC* knockout method based on the pT18sacB-sacB counterselection system. Short homologous arms were constructed by splice overlap extension PCR, and the knockout mutant Δ*fliC* was obtained through S17-1 λpir-mediated conjugation and sucrose counterselection. The mutant was verified by junction PCR, internal PCR, and sequencing. Using *Pinus massoniana* callus as the experimental material, we preliminarily compared the wild-type strain and the Δ*fliC* mutant in individual pathogenicity and in promoting complex infection by *B. xylophilus*. The Δ*fliC* mutant showed delayed symptom onset and lower disease severity than the wild type, and its ability to promote complex infection also appeared reduced. These results provide preliminary evidence that *fliC* contributes to symptom development in this callus assay. The established *fliC* knockout technique provides a methodological basis for further investigating the role of associated bacteria in pine wilt disease.

## Simple Summary

This study found that bacteria lacking the *fliC* gene showed slower symptom development and reduced disease severity on *Pinus massoniana* callus. Their capacity to assist pine wood nematodes in infecting pine tissues also appeared lower. These findings provide preliminary evidence that *fliC* contributes to symptom development in this assay. This work provides a methodological reference for further clarifying the role of associated bacteria in pine wilt disease.

## 1. Introduction

*Pinus massoniana* is a core native coniferous species in subtropical China, supporting timber production and resin tapping in southern China and sustaining plantation forestry [1,2]. In recent years, pine wilt disease has increasingly affected seedling survival, plantation establishment, and forest management in *P. massoniana* plantations, causing substantial losses and severely constraining the sustainable development of *P. massoniana* forests [3,4]. Callus tissue was used as a controlled early-interaction material because it is involved in wound responses [5], can be maintained under uniform conditions, and reduces host heterogeneity relative to intact seedlings or field-grown trees. It therefore provides a reproducible system for preliminary comparison of symptom development among treatments. Therefore, investigating the interaction between *P. massoniana* callus and pathogenic bacteria, and elucidating the functions of key pathogenicity genes, is of considerable theoretical and practical value for advancing molecular pathogenicity studies of forest diseases and constructing precision prevention and control systems.

Pine wilt disease is a devastating forest disease caused by *Bursaphelenchus xylophilus* infection, posing a serious threat to coniferous forest resources [3,4]. Three hypotheses have long been proposed regarding its pathogenic mechanism: the enzyme theory, the cavitation theory, and the toxin theory [6]. In recent years, the role of associated bacteria in the pathogenic process of *B. xylophilus* has received increasing attention [7], and research at the microbial level has provided new insights into the disease mechanism [8]. More than 290 bacterial species have been identified in association with *B. xylophilus* [9,10,11], and *Pseudomonas fluorescens* has been reported as a dominant associated bacterium [10,11]. *P. fluorescens* has also been reported to produce pine-cell-toxic substances and to act synergistically with *B. xylophilus* [7]. With the advancement of research, two bioactive compounds with pathogenic effects on suspension cells and seedlings of *Pinus thunbergii* were isolated from the culture broth of *P. fluorescens* [12,13,14]. In parallel, a flagellin toxin with a molecular mass of approximately 50 kDa was purified and identified as FliC from *P. fluorescens* [12,13,15]. Another study reported that an *fliC* engineered strain could restore pathogenicity to *P. thunbergii* seedlings [15]. These findings suggest that FliC flagellin may contribute to virulence-associated traits of *P. fluorescens*, but its specific role in the pathogenic process remains unclear. Construction of an *fliC* knockout mutant and comparison of its pathogenicity with the wild type are direct approaches to investigating the function of this gene. Accordingly, efficient knockout of *fliC* in *P. fluorescens* is a prerequisite for direct loss-of-function analysis of this gene.

Gene knockout is a classical approach for investigating gene function [16,17], but in Gram-negative bacteria such as *P. fluorescens*, the dense cell wall structure and poor outer membrane permeability result in generally low exogenous DNA transformation efficiency [18,19]. Conventional transformation methods are therefore difficult to apply directly, and gene knockout operations face substantial technical barriers. Various knockout strategies reported to date have shown limitations in this bacterium [20]. The CRISPR/Cas9 system is efficient and convenient, but Cas9 protein exerts potential toxicity on bacterial growth, requires inducible promoter regulation, and off-target effects are difficult to completely avoid [21]; the Red/ET recombination system requires prior expression of recombinase in the host cell [22], linear DNA fragments may be degraded by exonucleases [23], and system compatibility is suboptimal [24]; temperature-sensitive plasmid-mediated two-step homologous recombination is a traditional method, but it requires relatively long homologous arms, involves lengthy operation cycles, exhibits low screening efficiency, and suffers from prominent false-positive interference [25,26]. These limitations of existing methods have presented challenges for functional gene research. In recent years, the pT18sacB-based sacB-sucrose counterselection system has provided a new strategy for gene knockout in Gram-negative bacteria. This system utilizes the lethal effect of sucrose to achieve double-crossover screening without requiring a temperature-sensitive plasmid, and short homologous arms are sufficient to accomplish knockout. It features simple operation and a clean genetic background [20,25]. Therefore, establishing an *fliC* knockout technique applicable to *P. fluorescens* is of important methodological value and practical significance for further elucidating the pathogenic function of this gene.

Based on this, the present study used *P. fluorescens* as the target organism to construct a pT18sacB-based *fliC* knockout system. The Δ*fliC* mutant was obtained through conjugation and sacB counterselection. Using *P. massoniana* callus pathogenicity assays, we preliminarily evaluated the contribution of *fliC* to individual bacterial pathogenicity and in promoting complex infection by *B. xylophilus*. This study provides experimental evidence for clarifying the role of associated bacteria in the complex pathogenic mechanism of pine wilt disease.

## 2. Materials and Methods

### 2.1. Experimental Materials

**Figure 1:**
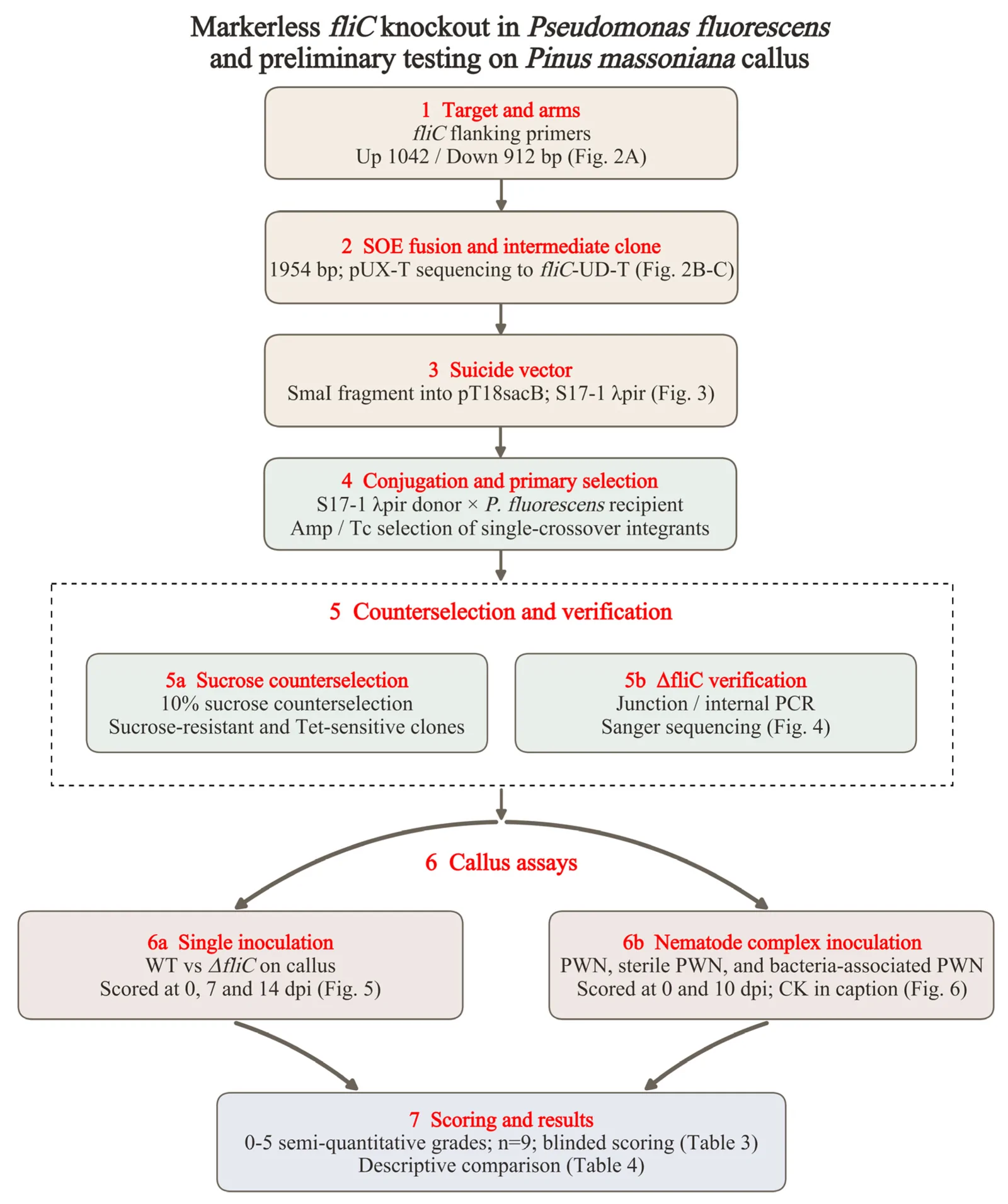
Graphical summary of *fliC* knockout and callus-based evaluation.

The *P. fluorescens* strain ATCC 13525 was used as the recipient strain for *fliC* knockout and subsequent *P. massoniana* callus pathogenicity evaluation (Figure 1), and was preserved at the Key Laboratory of Vector Biology and Pathogen Control of Zhejiang Province. *P. fluorescens* has been reported as a dominant associate on the surface of *B. xylophilus* [10,11]. Prior to the experiment, the bacterial stock was retrieved from a −80 °C freezer, streaked onto an LB agar plate without antibiotics, and incubated at 25 °C overnight. A single colony was picked and inoculated into LB liquid medium without antibiotics, then incubated at 25 °C to the logarithmic growth phase. *E. coli* DH5α competent cells were purchased from Hangzhou Baisai Biotechnology Co., Ltd. (Hangzhou, China), and were used for cloning of homologous arm fusion fragments and plasmid amplification. S17-1 λpir served as the donor strain for delivering the recombinant plasmid into *P. fluorescens*. The *fliC* knockout mutant Δ*fliC* was constructed in this study.

The plasmid system included pUX-T, pT18sacB, and the recombinant plasmids *fliC*-UD-T and *fliC*-pT18sacB constructed in this study. pUX-T was purchased from Hangzhou Baisai Biotechnology Co., Ltd. (Hangzhou, China) and was used for cloning of fused homologous arm fragments. pT18sacB carries tetracycline resistance and the sacB gene; the sacB gene causes host cell death in the presence of sucrose. pT18sacB cannot replicate autonomously in *P. fluorescens* and must be integrated into the genome through homologous recombination for stable maintenance. *B. xylophilus* was isolated from diseased *P. massoniana* wood in a previous study by our laboratory and was preserved at the Key Laboratory of Vector Biology and Pathogen Control of Zhejiang Province. Prior to the experiment, the nematodes were cultured on PDA plates with *Botrytis cinerea* as feed.

Antibiotic sensitivity testing was performed to determine the susceptibility of *P. fluorescens* to ampicillin (Amp), kanamycin (Kan), spectinomycin (Spc), chloramphenicol (Cm), gentamicin (Gen), tetracycline (Tc), and streptomycin (Str). The results showed that this strain was resistant to ampicillin and highly sensitive to tetracycline. The results are presented in Table 1.

PCR amplification primers were designed based on the *fliC* gene and its flanking sequences in the *P. fluorescens* genome, and were synthesized by Fengyuan Biotechnology Co., Ltd. The primer sequences and their uses are listed in Table 2.

The main reagents included 2× SuperPfu PCR mix, 2× Pfu PCR mix, 2× Taq PCR mix, 5× infusion mix, PCR product purification kit, pUX-T vector, DL2000 DNA marker, 2K Plus II DNA marker, and *E. coli* DH5α competent cells. Among these, 2K Plus II DNA marker was purchased from Beijing TransGen Biotech Co., Ltd. (Beijing, China), and all other reagents were purchased from Hangzhou Baisai Biotechnology Co., Ltd. (Hangzhou, China). FastDigest SmaI was purchased from Thermo Fisher Scientific (Waltham, MA, USA). Sequencing services were provided by Hangzhou Tsingke Biotechnology Co., Ltd. (Hangzhou, China).

The culture media included LB liquid medium (tryptone 10.0 g/L, yeast extract 5.0 g/L, NaCl 10.0 g/L) and LB solid medium (LB liquid medium supplemented with agarose 15.0 g/L), LB solid medium containing tetracycline, LB solid medium containing ampicillin and tetracycline, and LB solid medium containing 10% sucrose. Sucrose was added after sterilization. Potato dextrose agar (PDA) medium (glucose 20.0 g/L, agar powder 18.0 g/L, peeled potato 200.0 g/L) was used for *B. xylophilus* propagation. All media were sterilized at 121 °C and 0.1 MPa for 25 min.

The instruments used in this study included a thermal cycler (T100™, Bio-Rad, Hercules, CA, USA), a high-speed refrigerated centrifuge (1-16K, Sigma, St. Louis, MO, USA), a biochemical incubator (LRH-250F, Shanghai Yiheng, Shanghai, China), a gel documentation system (Gel Doc™, Bio-Rad, Hercules, CA, USA), an ultra-micro protein and nucleic acid analyzer (uLite+, BioDrop, Cambridge, UK), a clean bench (SW-CJ-2F, Shanghai Boxun, Shanghai, China), a constant-temperature shaking incubator (HZWY-2102C, Shanghai Zhicheng, Shanghai, China), a horizontal nucleic acid electrophoresis tank (DYCP-31DN, Beijing Liuyi, Beijing, China), and a vertical autoclave (2018N96, Shandong Shinva, Shandong, China).

### 2.2. fliC Gene Knockout Method

Based on the natural resistance of *P. fluorescens* to ampicillin and its high sensitivity to tetracycline, this study adopted the pT18sacB-sacB counterselection system to establish an *fliC* knockout method. Genomic DNA was extracted from *P. fluorescens*. Using genomic DNA as the template, PCR amplification was performed with primers *fliC*-up-F/*fliC*-up-R and *fliC*-down-F/*fliC*-down-R. The reaction program consisted of pre-denaturation at 94 °C for 5 min, followed by 30 cycles of denaturation at 94 °C for 30 s, annealing at 55 °C for 30 s, and extension at 72 °C for 40 s. The upstream homologous arm (1042 bp) and downstream homologous arm (912 bp) of *fliC* were obtained.

To generate the fused deletion fragment, the amplified products were purified and recovered, and splice overlap extension (SOE)-PCR was performed using primers *fliC*-up-F/*fliC*-down-R. The reaction program consisted of pre-denaturation at 94 °C for 5 min, 2 cycles of denaturation at 94 °C for 30 s, annealing at 50 °C for 30 s, and extension at 68 °C for 1 min, followed by 30 cycles of denaturation at 94 °C for 30 s, annealing at 55 °C for 30 s, and extension at 68 °C for 1 min. The fusion fragment (1954 bp) was obtained. The fusion fragment was ligated into the pUX-T vector and transformed into *E. coli* DH5α. Colony PCR was performed using primers M13-F(pUC)/M13-R(pUC) to screen positive clones, and the recombinant plasmid *fliC*-UD-T was obtained.

To construct the suicide knockout vector, the fusion fragment (1987 bp) was amplified from the *fliC*-UD-T plasmid using primers *fliC*-pT18-SmaI-F/*fliC*-pT18-SmaI-R. The pT18sacB plasmid was digested with SmaI (25 °C, 1 h). The amplified fragment was ligated to the digested vector by seamless cloning and transformed into *E. coli* S17-1 λpir. The transformants were plated on tetracycline-containing agar plates, and colony PCR was performed using primers M13-F(pUC)/M13-R(pUC) to identify positive clones. The recombinant knockout vector *fliC*-pT18sacB was obtained.

*E. coli* S17-1 λpir harboring *fliC*-pT18sacB was used as the donor strain, and *P. fluorescens* was used as the recipient strain for conjugation. Single colonies of the donor and recipient strains were inoculated into LB liquid medium (donor strain containing tetracycline) and LB liquid medium without antibiotics, respectively, and cultured to an OD_600_ of approximately 0.8. Aliquots of 700 μL from each culture were mixed, centrifuged to collect the cells, and placed on a sterilized nitrocellulose filter membrane at the center of an LB plate for overnight incubation. The filter membrane was washed with LB medium, diluted, and spread on Amp/Tc double-antibiotic plates. Single colonies were picked to confirm stable plasmid maintenance, yielding single-crossover integrants.

To obtain the markerless Δ*fliC* mutant, the single-crossover integrants were cultured on LB medium containing 10% sucrose. Clones that grew on sucrose plates but not on tetracycline plates were selected. Junction PCR was performed using primers *fliC*-JD-F/*fliC*-JD-R; the wild-type strain produced a 2519 bp product, while the knockout strain produced a 610 bp product. Internal PCR was performed using primers *fliC*-ter-F/*fliC*-ter-R; the wild-type strain produced a 1439 bp product, while no product was obtained from the knockout strain. The junction PCR products were subjected to Sanger sequencing to confirm deletion of the *fliC* coding region and correct ligation of the flanking sequences. The knockout strain confirmed by this three-level verification was designated Δ*fliC*. The verified Δ*fliC* strain was used for the subsequent pathogenicity evaluation.

### 2.3. Pathogenicity Evaluation Method

*P. massoniana* callus was obtained from the Research Institute of Subtropical Forestry, Chinese Academy of Forestry. The callus was subcultured and propagated on DCR medium supplemented with 6-BA 1 mg/L, 2,4-D 2 mg/L, sucrose 30 g/L, and agar 7 g/L. The pH was adjusted to 5.8. The culture temperature was 25 °C.

Healthy callus tissue with a milky white color and compact texture was selected for the infection experiment. Seven treatment groups were established: (1) wild-type *P. fluorescens* (WT) bacterial suspension; (2) *fliC* knockout mutant (Δ*fliC*) bacterial suspension; (3) wild-type *B. xylophilus* (PWN) suspension; (4) sterile *B. xylophilus* suspension; (5) *B. xylophilus* suspension associated with WT *P. fluorescens*; (6) *B. xylophilus* suspension associated with Δ*fliC P. fluorescens*; and (7) sterile water control (CK).

Preparation of bacterial suspensions: WT and Δ*fliC* strains were streaked onto LB plates without antibiotics and incubated at 25 °C for 48 h. Bacterial lawns were washed off with sterile physiological saline and vortexed to prepare bacterial suspensions. The concentration was adjusted to 10^5^ CFU/mL by plate counting for subsequent use.

Preparation of *B. xylophilus* suspension: The PWN used in this experiment was a strain previously isolated from diseased *P. massoniana* wood and preserved in our laboratory. After propagation on *B. cinerea* culture, fresh nematodes were collected using the modified Baermann funnel method, washed three times with sterile water, and the concentration was adjusted to approximately 1000 individuals/50 μL. Sterile *B. xylophilus* was obtained by surface sterilization with hydrogen peroxide: fresh nematodes were shaken in 30% H_2_O_2_ for 10 min, washed three times with sterile water, and inoculated onto LB plates. After incubation at 25 °C for 72 h with no visible colony growth, the nematodes were transferred to B. cinerea culture for propagation.

Preparation of the bacteria-nematode complex: Sterile *B. xylophilus* was mixed with equal volumes of WT or Δ*fliC* bacterial suspension and co-cultured at 27 °C with shaking for 12 h to allow association of the target bacteria with the nematode surface. The nematodes were then collected by centrifugation, washed three times with sterile water to remove free bacteria on the surface, and the nematode concentration was adjusted to approximately 1000 individuals/50 μL for subsequent use.

The infection experiment was conducted in a clean bench. Pre-cultured *P. massoniana* callus was transferred to fresh DCR medium plates. Each treatment was inoculated with 3 pieces of callus per independent experiment, and the experiment was repeated three times on different days, yielding 9 biological replicates per treatment. Bacterial and nematode suspensions were freshly and independently prepared for each independent experiment. Foreseeable sources of variation were controlled by using the same callus source and culture system, a uniform inoculum concentration, and consistent culture conditions. For the bacterial treatment groups (WT and Δ*fliC*), 0.4 mL of bacterial suspension (containing approximately 4 × 10^4^ CFU) was dropped onto the surface of each callus piece. For the nematode treatment groups (PWN, sterile nematodes, nematodes associated with WT, and nematodes associated with Δ*fliC*), 50 μL of nematode suspension (approximately 1000 individuals) was dropped onto each piece. The CK group received an equal volume of sterile water. The plates were placed in a light incubator at 25 °C for continued culture.

Photography time points were set as follows: the bacterial treatment groups (WT and Δ*fliC*) were photographed at 0 days post inoculation (dpi), 7 dpi, and 14 dpi; the nematode treatment groups (PWN and sterile nematodes) and the complex infection treatment groups (nematodes associated with WT and nematodes associated with Δ*fliC*) were photographed at 0 dpi and 10 dpi. Symptom changes were observed and recorded before each photography session. Note that bacterial treatments and nematode/complex treatments were assessed at different time points due to their distinct pathogenic dynamics.

A 0–5 ordinal symptom scoring system was used for semi-quantitative evaluation of pathogenic symptoms. The scoring criteria are shown in Table 3. Nine pieces of callus (3 pieces × 3 replicates) from each treatment group were scored independently by two researchers in a blinded manner. The results are presented as symptom grades to describe symptom progression among treatments. Because the endpoint was ordinal and semi-quantitative, biological variability was controlled through the replicate structure and blinded scoring but was not estimated by variance modeling.

## 3. Results

### 3.1. Construction and Verification of the fliC Knockout Mutant

Using *P*. *fluorescens* genomic DNA as the template, the upstream (1042 bp) and downstream (912 bp) homologous arms of *fliC* were amplified by PCR. The arms were fused to a 1954 bp fragment by SOE-PCR and cloned into pUX-T to construct *fliC*-UD-T. The fusion fragment with SmaI arms (1987 bp) was then amplified and ligated to linearized pT18sacB by seamless cloning to construct *fliC*-pT18sacB. Through conjugation and sacB counterselection, the *fliC* knockout mutant Δ*fliC* was obtained and verified at three levels: junction PCR, internal PCR, and sequencing.

**Figure 2:**
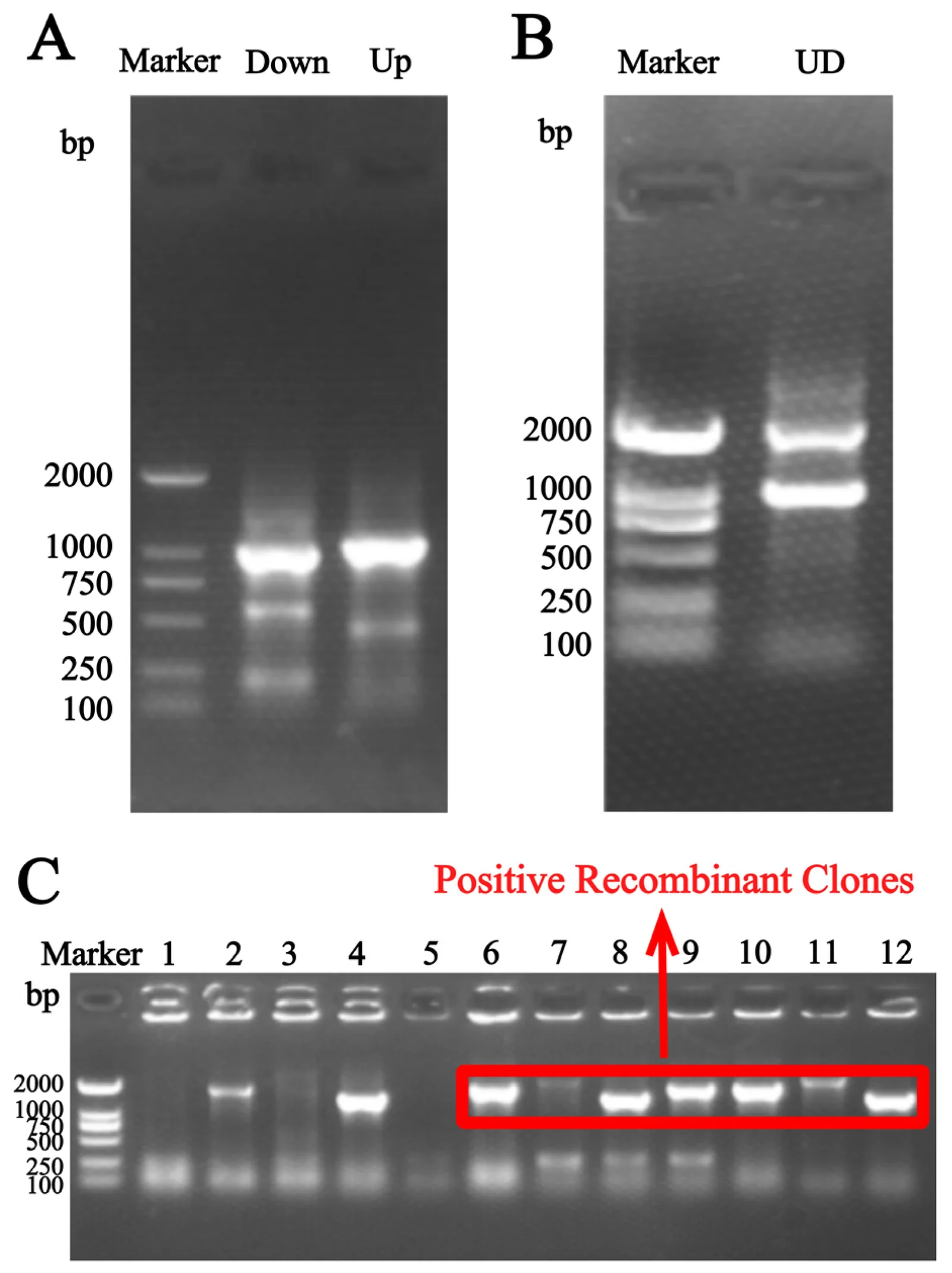
Construction of Homologous Arms and Verification of the Intermediate Vector: (**A**) PCR amplification electrophoresis of the upstream and downstream homologous arms of *fliC*. Lane M: DNA marker; lane up: upstream homologous arm (1042 bp); lane down: downstream homologous arm (912 bp). (**B**) PCR amplification electrophoresis of the splice overlap extension fusion fragment. Lane M: DNA marker; lane UD: fusion fragment (1954 bp). (**C**) Colony PCR identification of pUX-T positive clones. Lane M: DNA marker; lanes 1–12: randomly selected clones.

The amplification and fusion results of the homologous arms are shown in Figure 2. Using *P. fluorescens* genomic DNA as the template, the upstream and downstream homologous arms were amplified with primers *fliC*-up-F/*fliC*-up-R and *fliC*-down-F/*fliC*-down-R, respectively. Agarose gel electrophoresis (1%) showed clear bands of approximately 1000 bp for the upstream homologous arm and approximately 900 bp for the downstream homologous arm (Figure 2A), consistent with the expected sizes of 1042 bp and 912 bp. The purified upstream and downstream homologous arms were mixed at equimolar ratios and used as templates for SOE-PCR fusion with outer primers *fliC*-up-F/*fliC*-down-R. The electrophoresis result showed a single bright band of approximately 2000 bp for the fusion fragment (Figure 2B), consistent with the expected size calculated from the primer and flanking sequences, indicating successful fusion of the homologous arms by overlap extension. The fusion fragment was ligated into the pUX-T vector and transformed into *E. coli* DH5α. Twelve clones were randomly selected for colony PCR identification using universal primers M13-F(pUC)/M13-R(pUC). Positive clones were identified among the twelve clones screened (Figure 2C), indicating insertion of the fusion fragment into the pUX-T vector. The positive clones were subjected to scale-up culture and plasmid extraction, and the intermediate recombinant plasmid *fliC*-UD-T was confirmed by sequencing.

**Figure 3:**
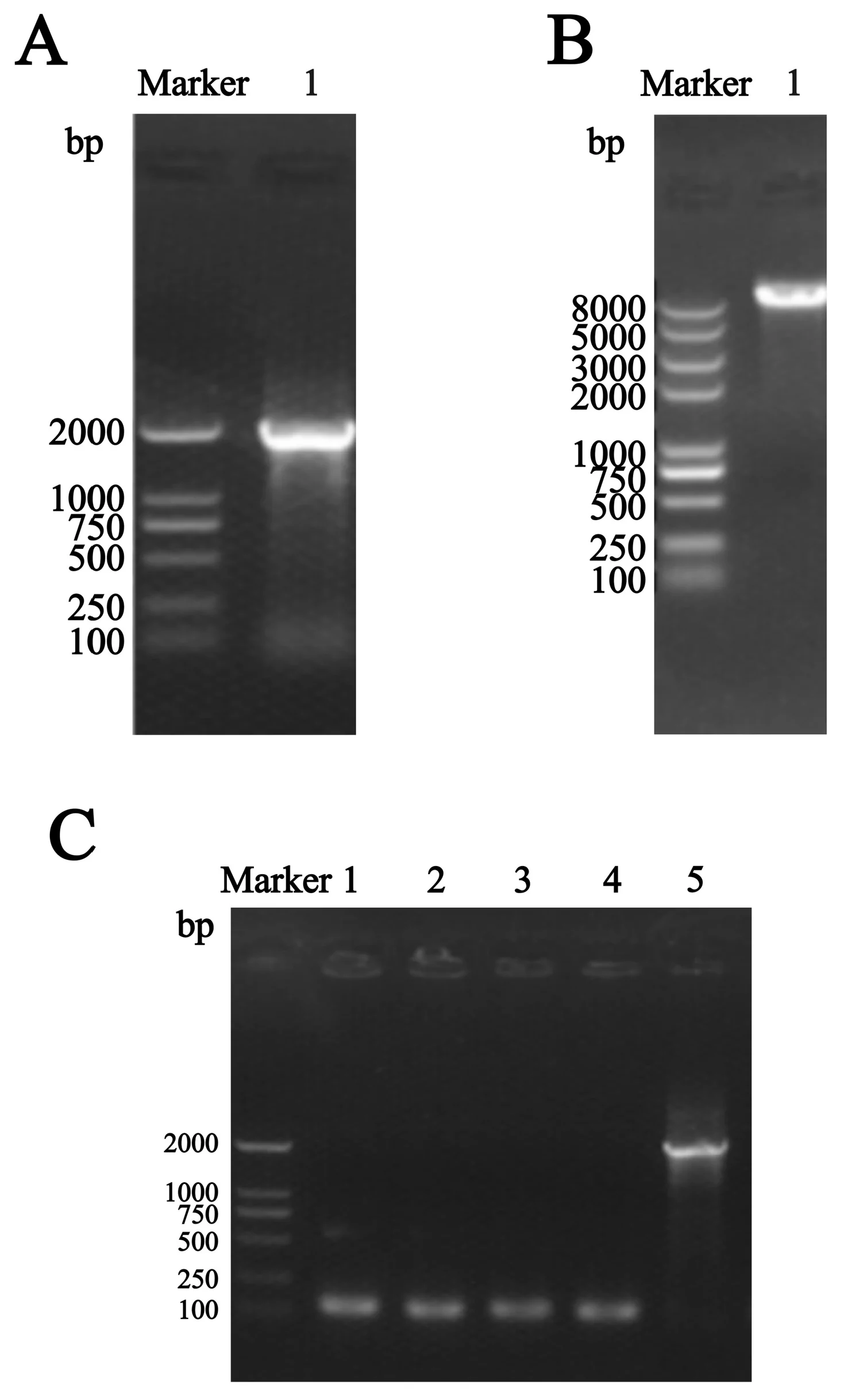
Construction and Verification of the Suicide Knockout Vector *fliC*-pT18sacB: (**A**) PCR amplification electrophoresis of the fusion fragment with vector homologous arms. Lane M: DNA marker; lane 1: fusion fragment (1987 bp). (**B**) SmaI single-enzyme digestion electrophoresis of pT18sacB. Lane M: DNA marker; lane 1: digested product (5721 bp). (**C**) Colony PCR identification of positive clones of the knockout vector. Lane M: DNA marker; lanes 1–5: randomly selected clones.

The construction and verification of the suicide knockout vector are shown in Figure 3. Using the *fliC*-UD-T plasmid as the template, the fusion fragment with vector homologous arms was amplified with primers *fliC*-pT18-SmaI-F/*fliC*-pT18-SmaI-R. The electrophoresis result showed a specific band of approximately 2000 bp (Figure 3A), consistent with the expected 1987 bp. Meanwhile, the pT18sacB plasmid was digested with SmaI, and agarose gel electrophoresis (1%) showed a linearized band of approximately 5700 bp (Figure 3B), consistent with the expected 5721 bp, confirming linearization of the vector. The recovered fusion fragment and linearized pT18sacB were ligated at a 3:1 molar ratio by seamless cloning and transformed into *E. coli* S17-1 λpir. The transformants were plated on LB plates containing Tc, and 5 clones were randomly selected for colony PCR identification. Positive clones were identified among the five randomly selected clones (Figure 3C), indicating insertion of the fusion fragment into the pT18sacB vector. Plasmid extraction and restriction enzyme digestion confirmed correct construction of the recombinant vector, which was named *fliC*-pT18sacB.

The *fliC*-pT18sacB vector was transferred from the *E. coli* S17-1 λpir donor strain to the *P. fluorescens* recipient strain by filter membrane conjugation. The bacteria were plated on Amp/Tc double-antibiotic plates and incubated at 25 °C for 48 h to obtain single-crossover integrants. The single-crossover integrants were plated on LB plates containing 10% sucrose for counterselection. After incubation at 25 °C for 72 h, candidate clones that grew well on sucrose plates but not on Tc plates were selected.

**Figure 4:**
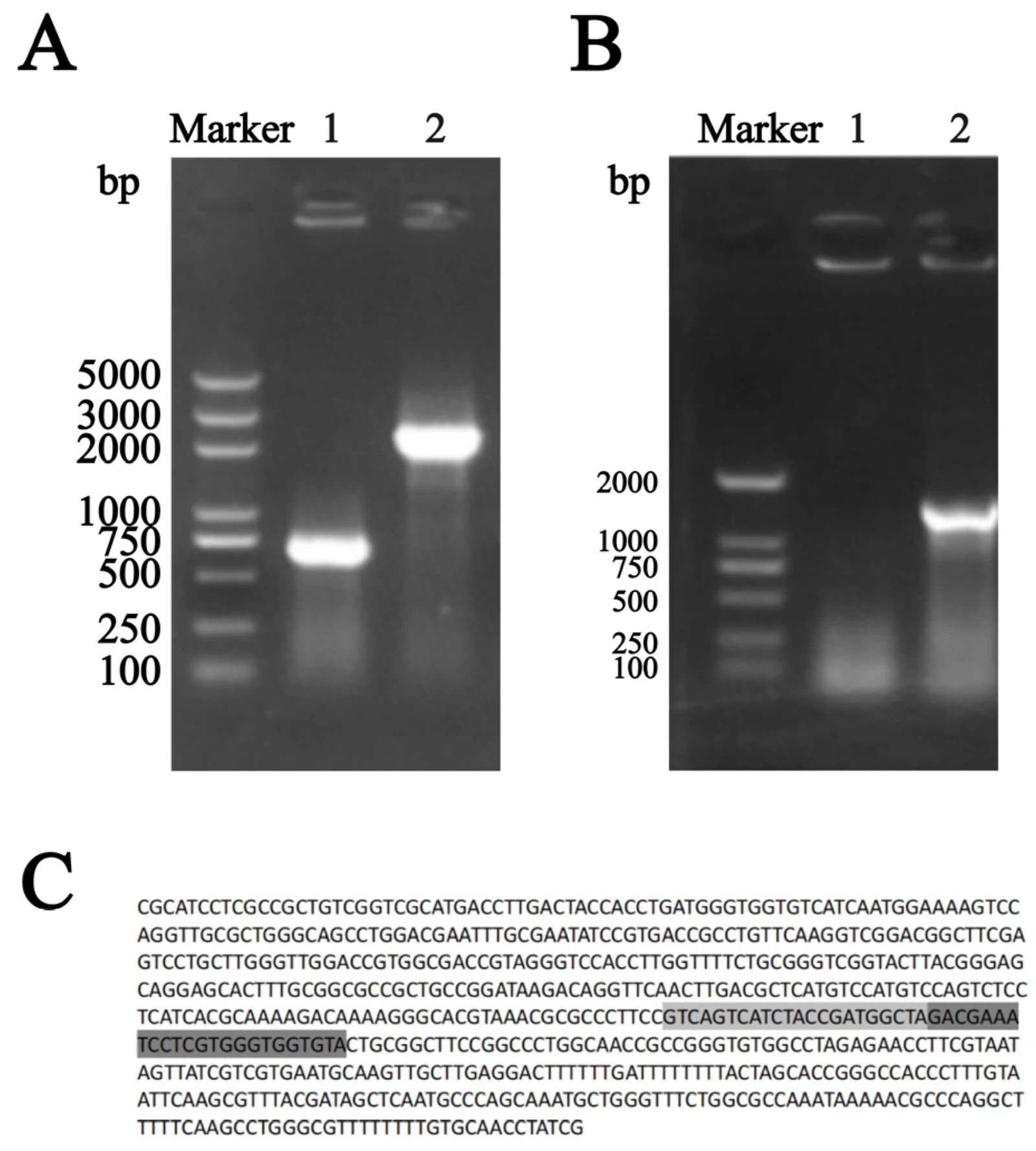
Three-Level Verification of the *fliC* Knockout Mutant: (**A**) Junction PCR electrophoresis with primers *fliC*-JD-F/R. Lane M: DNA marker; lane 1: knockout strain (610 bp); lane 2: wild-type strain (2519 bp). (**B**) Internal PCR electrophoresis with primers *fliC*-ter-F/R. Lane M: DNA marker; lane 1: knockout strain (no amplification product); lane 2: wild-type strain (1439 bp). (**C**) Representative Sanger sequencing result of the knockout strain.

The three-level verification results of the knockout mutant are shown in Figure 4. Junction PCR was performed with primers *fliC*-JD-F/*fliC*-JD-R, which span the genomic regions flanking the *fliC* coding region. The wild-type (WT) strain showed an amplified band of approximately 2500 bp (Figure 4A), consistent with the expected 2519 bp. The candidate knockout strain (Δ*fliC*) showed a markedly shortened band of approximately 600 bp (Figure 4A), consistent with the expected 610 bp, indicating that the *fliC* coding region had been deleted in a large fragment. Internal PCR was performed with primers *fliC*-ter-F/*fliC*-ter-R, which are located within the *fliC* coding region. The WT strain amplified a specific band of approximately 1400 bp (Figure 4B), consistent with the expected 1439 bp, while the candidate knockout strain showed no amplification product (Figure 4B), further confirming that the internal sequence of the *fliC* gene was completely absent. The junction PCR products were sent to Hangzhou Tsingke Biotechnology Co., Ltd. for Sanger sequencing. The Sanger sequencing result showed that the knockout strain exhibited sequence truncation at the expected *fliC* coding region, with correct ligation of the upstream and downstream genomic sequences and no base mutations or residual sequences (Figure 4C), confirming that the *fliC* gene had been knocked out. Based on these three-level verification results, the *fliC* knockout mutant Δ*fliC* was obtained.

### 3.2. Pathogenicity Differences Between the Wild-Type and Knockout Strains on Pinus massoniana Callus

**Figure 5:**
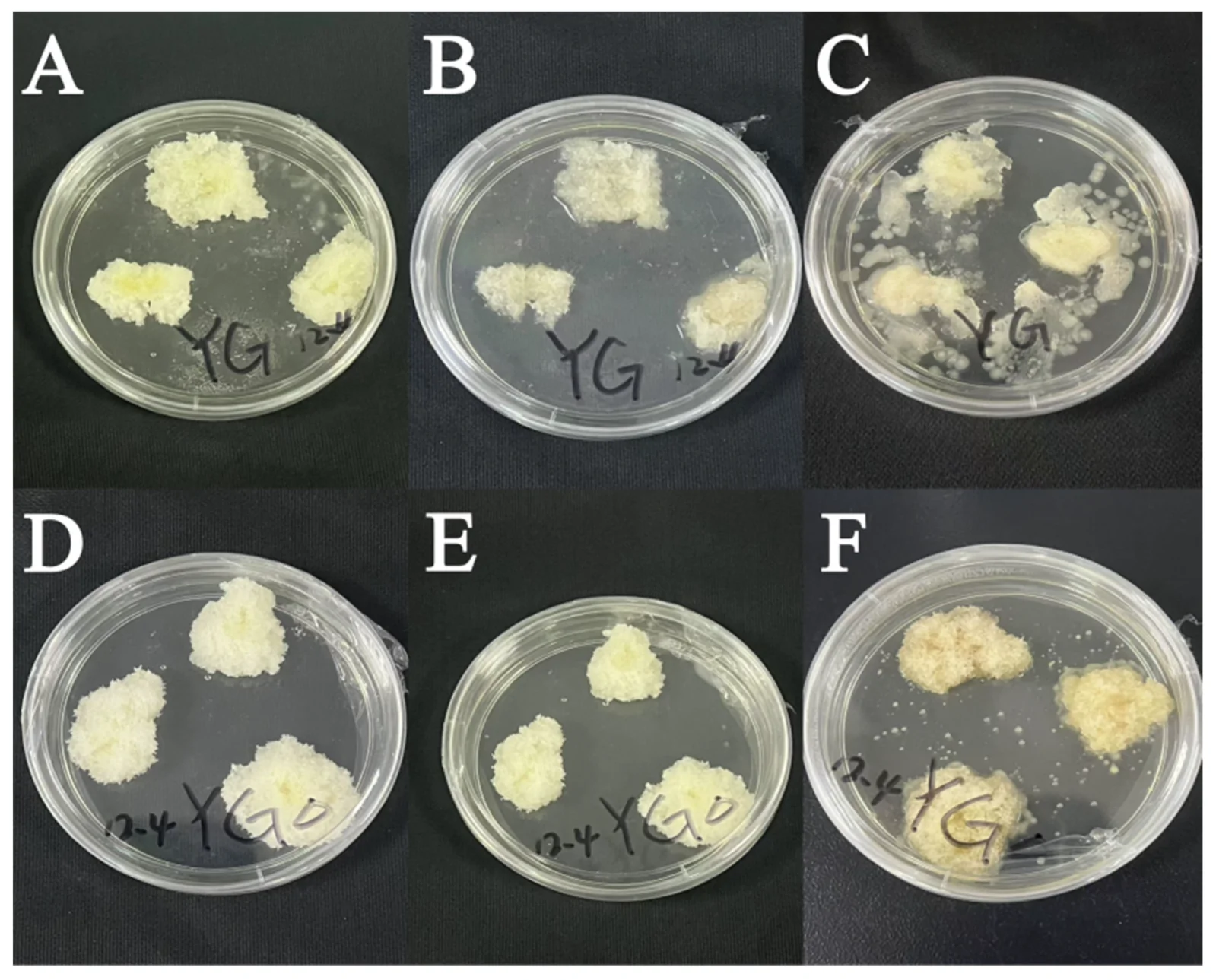
Pathogenicity Differences between the Wild-Type and Knockout Strains on *Pinus massoniana* Callus: (**A**,**D**) Before inoculation (0 dpi); (**B**) WT 7 dpi; (**C**) WT 14 dpi; (**E**) Δ*fliC* 7 dpi; (**F**) Δ*fliC* 14 dpi.

To investigate the effect of the FliC flagellin gene on the pathogenic function of *P*. *fluorescens*, the wild-type strain WT and the *fliC* knockout mutant Δ*fliC* were used to infect *P. massoniana* callus cultured in vitro, and symptom changes were observed and recorded at 0, 7, and 14 dpi. The results are shown in Figure 5.

Before inoculation (0 dpi), callus tissue in all treatment groups was milky white to pale yellow, with compact texture and intact clump structure, showing no obvious disease symptoms (Figure 5A,D). Seven dpi after inoculation with the WT bacterial suspension, the callus surface showed obvious wrinkling, marginal browning, slight water-soaking diffusion on the medium, and the clump structure became loose (Figure 5B). At 14 dpi, callus in the WT treatment group completely lost its original clump morphology, turned dark brown in color, showed large water-soaking halos on the medium, was covered with obvious bacterial lawns on the surface, and the cellular structure was completely disintegrated (Figure 5C). In contrast, callus in the Δ*fliC* treatment group showed almost no change in appearance at 7 dpi compared with 0 dpi, with normal color, intact clump structure, and no water-soaking (Figure 5E). At 14 dpi, callus in the Δ*fliC* treatment group only exhibited slight browning, a small amount of water-soaking at the edges, largely maintained original morphology, and only trace colony growth was observed on the medium (Figure 5F). Meanwhile, the sterile water control group grew normally at 14 dpi, with milky white color and compact structure. These results suggest that *fliC* deletion is associated with reduced symptom development of *P. fluorescens* on *P. massoniana* callus.

**Figure 6:**
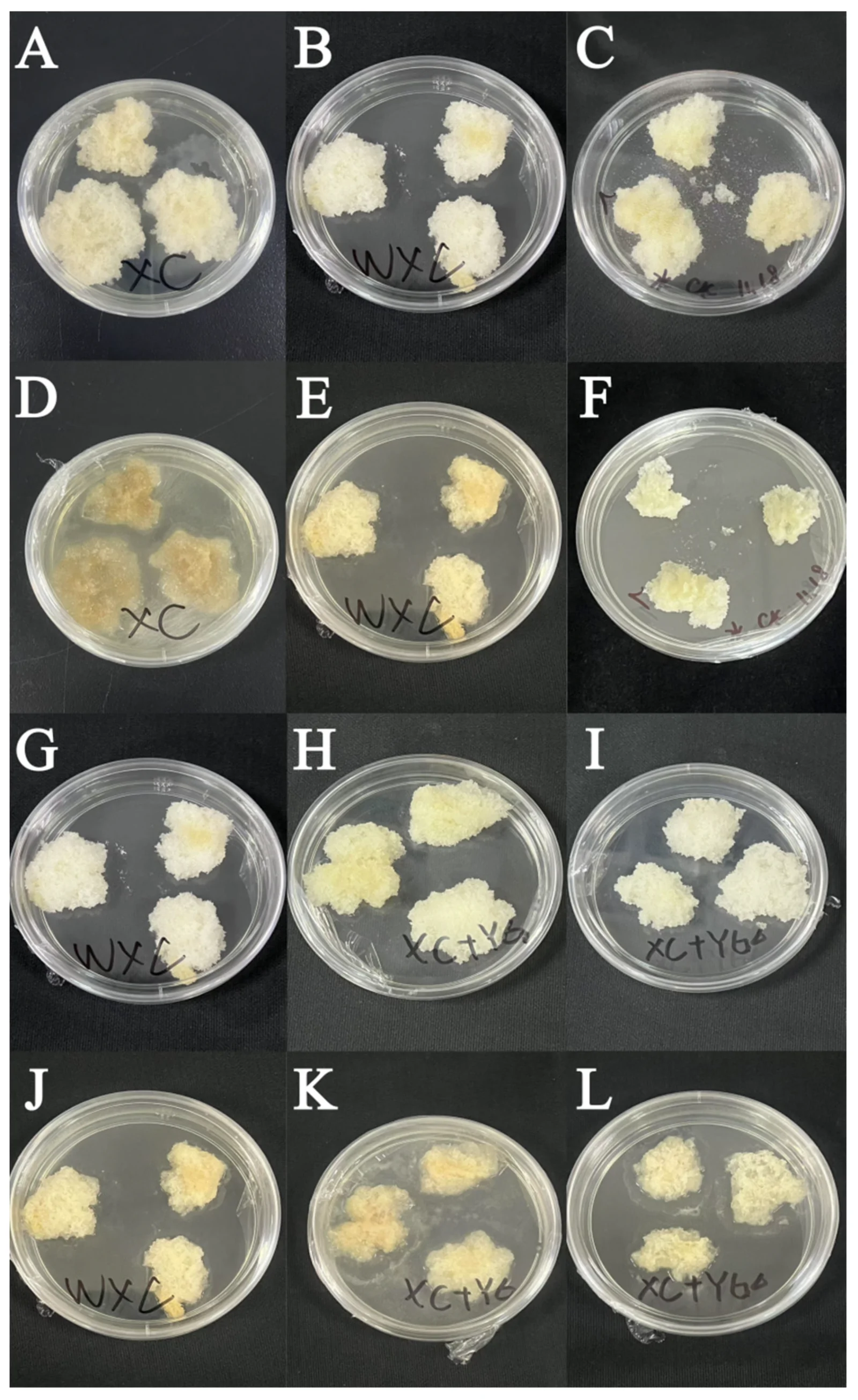
Effect of *fliC* Deletion on *Pseudomonas fluorescens*-Promoted Complex Infection by *Bursaphelenchus xylophilus*: (**A**,**D**) Wild-type *B. xylophilus* (PWN) at 0 and 10 dpi; (**B**,**E**) Sterile *B. xylophilus* at 0 and 10 dpi (single treatment); (**C**,**F**) Sterile water control at 0 and 10 dpi; (**G**,**J**) Sterile *B. xylophilus* at 0 and 10 dpi (complex infection control); (**H**,**K**) PWN associated with WT *P. fluorescens* at 0 and 10 dpi; (**I**,**L**) PWN associated with Δ*fliC P. fluorescens* at 0 and 10 dpi.

The results of single infection by *B. xylophilus* and sterile *B. xylophilus* are shown in Figure 6A–F. Before inoculation (0 dpi), callus tissue in the wild-type *B. xylophilus* (PWN), sterile *B. xylophilus*, and sterile water treatment groups was in normal condition (Figure 6A–C). Ten dpi after inoculation, callus in the PWN treatment group showed severe browning, complete collapse of the clump structure, and large-area water-soaking on the medium (Figure 6D). The sterile *B. xylophilus* treatment group only showed slight yellowing at 10 dpi, the clump structure remained largely intact, cells grew normally, and no obvious water-soaking was observed (Figure 6E). The sterile water control group showed no visible changes at 10 dpi (Figure 6F).

The results of complex infection by *B. xylophilus* associated with *P. fluorescens* are shown in Figure 6G–L. Before inoculation, callus tissue in all treatment groups was in good condition (Figure 6G–I). At 10 dpi, callus in the *B. xylophilus* treatment group associated with WT *P. fluorescens* showed severe browning, large-area water-soaking diffusion on the medium, and the originally compact and swollen clump structure became loose, with cell growth completely blocked (Figure 6K). At 10 dpi, callus in the *B. xylophilus* treatment group associated with Δ*fliC P. fluorescens* showed slight browning, water-soaking was observable, and the clump exhibited some morphological changes, but the damage was less severe than that in the WT-associated treatment group (Figure 6L). The sterile *B. xylophilus* treatment group at 10 dpi showed performance consistent with Figure 6E, with only slight yellowing and largely intact structure (Figure 6J).

The symptom grades of all treatment groups are summarized in Table 4. In the bacterial treatments, WT *P. fluorescens* reached a moderate grade at 7 dpi and a necrotic grade at 14 dpi, whereas the Δ*fliC* mutant remained largely normal at 7 dpi and showed only slight symptoms at 14 dpi. In the nematode and complex treatments at 10 dpi, the PWN associated with WT *P. fluorescens* group showed the most severe symptoms, wild-type PWN alone was intermediate, PWN associated with Δ*fliC P. fluorescens* was milder, and sterile *B. xylophilus* and the sterile water control showed the mildest or no symptoms.

## 4. Discussion

### 4.1. Technical Advantages of the Knockout Method

Figure 2, Figure 3 and Figure 4 summarize the knockout and verification workflow, with primer information and antibiotic-selection basis provided in Table 1 and Table 2. In line with this workflow, the Δ*fliC* mutant showed a 1909 bp shortening of the junction PCR product, no internal *fliC* amplification, and correct ligation of the upstream and downstream genomic sequences by Sanger sequencing, together supporting deletion of the target coding region [27].

A multi-level progressive verification strategy was adopted to ensure the reliability of the genetic background [25]. Junction PCR reflects the large-fragment deletion of the *fliC* coding region from the perspective of fragment length. Internal PCR corroborates the deletion of *fliC* from the perspective of target gene presence. Sanger sequencing confirms the nucleotide composition of the ligation site at the sequence level. These three approaches collectively provide evidence of *fliC* deletion from different angles. Shortening of the junction PCR product may correspond to complete deletion, partial deletion, or rearrangement [28]. The absence of an internal PCR product may result from gene deletion or from interference by PCR inhibition factors [29]. A single dimension cannot independently exclude these alternative explanations. The combined and consistent results of all three approaches substantially improve the credibility of the genetic background of the knockout strain. This is particularly important for subsequent pathogenicity experiments, because unexpected mutations or vector remnants in the knockout strain may independently affect pathogenic performance, thereby interfering with the assessment of the *fliC* functional deficit effect.

The knockout approach eliminates the introduction of exogenous resistance markers [30]. The Δ*fliC* mutant genome contains no antibiotic resistance genes, avoiding potential interference from continuous expression of resistance genes that may activate host defense signals or alter host physiological status. It also excludes polar effects of the resistance expression cassette on neighboring genes [31]. These features reduce interference from resistance markers, but they cannot fully exclude polar effects on adjacent genes or unknown mutations. Therefore, the symptom differences observed between WT and Δ*fliC* are more likely to be associated with *fliC* deletion.

From a methodological perspective, this study provides a reference example for applying the pT18sacB-sacB counterselection strategy in *P. fluorescens*. Short homologous arms of approximately 1 kb reduce the difficulty of primer design and PCR operation, double-antibiotic screening together with sucrose counterselection generates clear selection pressure, and the three-level verification system provides a framework for confirming knockout strains. Together, these features are consistent with the intended advantage of sacB–sucrose counterselection in providing a cleaner genetic background for subsequent gene-function analysis in Gram-negative bacteria.

### 4.2. Effect of fliC Deletion on Individual Bacterial Pathogenicity

Figure 5 and Table 4 show that, on *P. massoniana* callus, WT produced obvious symptoms at 7 dpi and complete necrosis and disintegration at 14 dpi, whereas Δ*fliC* showed almost no symptoms at 7 dpi and only slight browning at 14 dpi, indicating that *fliC* deletion was associated with delayed symptom onset and reduced disease severity in this assay. Because bacterial growth and fitness were not measured, the contribution of possible growth defects to this milder phenotype cannot be excluded. Within these limits, the approximately one-week delay in symptom onset suggests that loss of *fliC* function may have slowed the infection process in host tissue [32]. The retention of slight damage at 14 dpi also indicates that Δ*fliC* did not lose all pathogenic capacity [32]. Thus, the pathogenicity of *P. fluorescens* is unlikely to be determined by a single factor; *fliC* more plausibly contributes to the rate and extent of symptom development, while other virulence factors may account for basal damage [33].

The faster symptom onset of WT is consistent with reports linking flagellar integrity to bacterial motility and chemotaxis [34], through which more motile wild-type cells may reach host tissue surfaces earlier and establish infection [35]. However, swimming, swarming, chemotaxis, biofilm formation, bacterial attachment, and colonization were not measured in the present study, and whether the flagellar structure or its components directly participate in host-cell damage cannot be determined from the current phenotypic data alone. The retention of weak pathogenicity in Δ*fliC* supports the interpretation that *fliC* mainly contributes to infection speed, but this cannot be regarded as the only explanation. It is also possible that *fliC* simultaneously participates in direct toxicity, but this contribution is partially masked by the presence of other virulence factors [33]. Without swimming, swarming, chemotaxis, biofilm, attachment, and colonization data, the relative contributions of motility, surface association, and other virulence functions cannot be distinguished in this study.

### 4.3. Effect of fliC Deletion on Complex Infection by Bursaphelenchus xylophilus

Figure 6 and Table 3 and Table 4 show a descriptive trend at 10 dpi, with symptom severity decreasing from *B. xylophilus* associated with WT *P. fluorescens* to wild-type *B. xylophilus* alone, then to *B. xylophilus* associated with Δ*fliC*, and finally to sterile *B. xylophilus*. This trend suggests that the presence of *P. fluorescens* was associated with enhanced symptom development of *B. xylophilus* on *P. massoniana* callus, and that this enhancement was weaker but not abolished after *fliC* deletion. Because the Δ*fliC*-associated complex treatment was less severe than both the WT-associated complex treatment and wild-type *B. xylophilus* alone, *fliC* may contribute to the *P. fluorescens* mediated enhancement of *B. xylophilus* infection. However, the remaining symptoms in the Δ*fliC* associated group indicate that *fliC* was not the only contributing factor and that other bacterial mechanisms may also assist nematode infection.

Sterile *B. xylophilus* alone caused only slight symptoms, whereas the WT bacteria-associated nematode group showed necrotic symptoms. This pattern agrees with background findings that associated *P. fluorescens* can contribute to pine cell damage and act synergistically with *B. xylophilus* [7]. It also indicates that *B. xylophilus* itself retains certain infection potential, but its pathogenic effect was relatively limited under the present conditions [4,36]. When the individual bacterial treatment and complex infection results are considered together, *fliC* deletion was accompanied by later symptom onset and lower disease severity, suggesting that *fliC* may be involved in promoting infection. However, Δ*fliC* did not completely lose pathogenic or infection-promoting capacity in either scenario. This observation raises the possibility that other pathogenic factors may partially compensate for *fliC* loss [33], but this possibility was not tested at the transcriptional or protein level. Thus, *fliC* should be considered a contributory factor rather than the sole determinant in these assays.

### 4.4. Research Limitations and Prospects

This study has several limitations. First, a complemented Δ*fliC* strain was not constructed, and whole-genome resequencing of the mutant was not performed. Therefore, unintended mutations and polar effects on adjacent genes cannot be fully excluded, and the present phenotype should be interpreted as an association rather than definitive proof of *fliC*-specific causality. Second, pathogenicity was evaluated using an in vitro callus system, and swimming, swarming, chemotaxis, biofilm formation, bacterial attachment, and colonization on callus or nematode surfaces were not directly measured. Bacterial growth curves and fitness were also not assessed; therefore, a fitness cost of *fliC* deletion cannot be excluded as a partial contributor to the reduced symptoms. Symptom evaluation was based on semi-quantitative morphological grading, and objective quantitative measurements such as lesion area, electrolyte leakage, and bacterial load were not obtained. The replication scale was also limited for quantitative inference, and batch-to-batch biological variability was not modeled because no continuous objective endpoint was available. Finally, *fliC* transcript and FliC protein levels, as well as the expression of other virulence-related genes, were not quantified, and RNA sequencing or proteomic analyses were not performed; therefore, expression-level polar effects and compensatory regulation can neither be excluded nor confirmed. Future work should increase independent biological replicates, restore *fliC* in the clean Δ*fliC* background, and measure these functional phenotypes. Future quantitative endpoints will be analyzed using pre-specified statistical methods with appropriate multiple-comparison correction. These assays should be further verified in potted pine seedlings and, where feasible, in more intact host systems that better approximate natural infection, together with the functional division among other members of the flagellar gene family.

## 5. Conclusions

This study used *P. fluorescens* as the target organism and established an *fliC* knockout method based on the pT18sacB-sacB-sucrose counterselection system. We preliminarily compared the individual pathogenicity of the wild-type strain and the Δ*fliC* mutant on *P. massoniana* callus, as well as their ability to promote complex infection by *B. xylophilus*. The results showed that *fliC* deletion was associated with reduced symptom development and a lower capacity to promote complex infection in this assay. These findings provide preliminary evidence that *fliC* contributes to symptom development in this assay, although the conclusion is limited by the callus system, the descriptive symptom endpoint, and the lack of complementation and whole-plant validation. Even with these limits, the established knockout platform may support functional gene studies in *P. fluorescens* and further analyses of bacteria–nematode–pine interactions, and future complementation and whole-plant studies are encouraged to refine the role of *fliC*.

## Figures and Tables

**Table 1:** Antibiotic sensitivity of *Pseudomonas fluorescens*.

Antibiotic	Abbreviation	Category	Sensitivity
Ampicillin	Amp	β-lactam	Resistant
Kanamycin	Kan	Aminoglycoside	Sensitive
Spectinomycin	Spc	Aminocyclitol	Sensitive
Chloramphenicol	Cm	Amphenicol	Sensitive
Gentamicin	Gen	Aminoglycoside	Sensitive
Tetracycline	Tc	Tetracycline	Highly sensitive
Streptomycin	Str	Aminoglycoside	Sensitive

**Table 2:** List of primers used in this study.

Primer Name	Sequence (5′-3′)	Purpose	Expected Product
*fliC*-up-F	TGCTGTATTGCGCGTTGATCGAG	Upstream homologous arm amplification	1042 bp
*fliC*-up-R	ACCCACGAGGATTTCGTCTAGCCATCGGTAGATGACTGAC	Upstream homologous arm amplification	-
*fliC*-down-F	CATCTACCGATGGCTAGACGAAATCCTCGTGGGTGGTGTA	Downstream homologous arm amplification	912 bp
*fliC*-down-R	ATCGACGCGCTGATCGTTGTGAC	Downstream homologous arm amplification	-
*fliC*-pT18-SmaI-F	CGAATTCGAGCTCGGTATGCTGTATTGCGCGTTGATCGAG	Fusion fragment amplification (with SmaI arm)	1987 bp
*fliC*-pT18-SmaI-R	TCTAGAGGATCCCCGGGATCGACGCGCTGATCGTTGTGAC	Fusion fragment amplification (with SmaI arm)	-
*fliC*-ter-F	AGCAGCTTCAGTACAGCGGAAGG	Internal PCR secondary identification of knockout strain	WT: 1439 bp; Δ*fliC*: 0 bp
*fliC*-ter-R	ATGGCATTAACAGTAAACACCAA	Internal PCR secondary identification of knockout strain	-
*fliC*-JD-F	GTTTCAACGCAGTTTCCGAAGGA	Junction PCR primary identification of knockout strain	WT: 2519 bp; Δ*fliC*: 610 bp
*fliC*-JD-R	TCCGTGATGCCTTGATAGGTTGC	Junction PCR primary identification of knockout strain	-
M13-F(pUC)	CCCAGTCACGACGTTGTAAAACG	Positive clone screening	-
M13-R(pUC)	AGCGGATAACAATTTCACACAGG	Positive clone screening	-

**Table 3:** Scoring criteria for pathogenic symptoms on *Pinus massoniana* callus.

Score	Grade	Color Change	Water-Soaking	Tissue Structure
0	Normal	Milky white to pale yellow, normal color	No water-soaking	Clumped, tightly swollen
1	Very slight	Slight yellowing	No obvious water-soaking	Clump structure largely maintained
2	Slight	Local browning or yellowing	Slight water-soaking	Clump slightly loose
3	Moderate	Obvious browning	Obvious water-soaking	Structure beginning to loosen
4	Severe	Severe browning	Large-area water-soaking	Clump loose and collapsed
5	Necrosis	Dark brown to black necrosis	Large-area water-soaking spreading to the medium	Clump collapsed and original structure lost

**Table 4:** Semi-quantitative symptom grades of *Pinus massoniana* callus across treatment groups.

Treatment Group	0 dpi	7 dpi	10 dpi	14 dpi
WT *Pseudomonas fluorescens*	0	3	-	5
Δ*fliC Pseudomonas fluorescens*	0	0	-	2
Wild-type *Bursaphelenchus xylophilus* (PWN)	0	-	4	-
Sterile *B. xylophilus*	0	-	1	-
PWN associated with WT *P. fluorescens*	0	-	5	-
PWN associated with Δ*fliC P. fluorescens*	0	-	3	-
Sterile water control (CK)	0	0	0	0

Note: Symptom grades were assigned according to the criteria in Table 3 and are ordinal semi-quantitative descriptors. For each treatment and time point, the grade shown was consistent across the nine biological replicates and the two independent scorers. Bacterial treatments and nematode/complex treatments were recorded at different time points and are not directly compared across assays.

## Data Availability

The original contributions presented in this study are included in the article. Further inquiries can be directed to the corresponding author.
